# Regulation of Pyroptosis by ncRNA: A Novel Research Direction

**DOI:** 10.3389/fcell.2022.840576

**Published:** 2022-03-28

**Authors:** Liyuan Gao, Zhitao Jiang, Yi Han, Yang Li, Xiang Yang

**Affiliations:** ^1^ Zhangjiagang TCM Hospital Affiliated to Nanjing University of Chinese Medicine, Suzhou, China; ^2^ Jiangsu Key Laboratory for Pharmacology and Safety Evaluation of Chinese Materia Medica, Nanjing University of Chinese Medicine, Nanjing, China

**Keywords:** inflammasomes, gasdermin, non-coding RNA, mechanism, pyroptosis

## Abstract

Pyroptosis is a novel form of programmed cell death (PCD), which is characterized by DNA fragmentation, chromatin condensation, cell swelling and leakage of cell contents. The process of pyroptosis is performed by certain inflammasome and executor gasdermin family member. Previous researches have manifested that pyroptosis is closely related to human diseases (such as inflammatory diseases) and malignant tumors, while the regulation mechanism of pyroptosis is not yet clear. Non-coding RNA (ncRNA) such as microRNA (miRNA), long non-coding RNA (lncRNA) and circular RNA (circRNA) have been widely identified in the genome of eukaryotes and played a paramount role in the development of cell function and fate after transcription. Accumulating evidences support the importance of ncRNA biology in the hallmarks of pyroptosis. However, the associations between ncRNA and pyroptosis are rarely reviewed. In this review, we are trying to summarize the regulation and function of ncRNA in cell pyroptosis, which provides a new research direction and ideas for the study of pyroptosis in different diseases.

## Introduction

Pyroptosis is a novel form of programmed cell death (PCD) plays a pivotal role in the survival and development of organisms, and it is also a way of self-protection (Bedoui et al., 2020). Apoptosis is the first PCD type to be discovered (D'Arcy, 2019), and the others including pyroptosis, necrosis, ferroptosis, autophagic cell death and paraptosis are gradually being revealed (Lee et al., 2016). Their main characteristics and differences are presented in Table 1. In 2005, Susan L. Fink (Fink and Cookson, 2005) et al. firstly dug out and defined the phenomenon of cell pyroptosis. Then pyroptosis is well-known as cell inflammatory necrosis (Xia et al., 2019), and it has been widely recognized as a new member of PCD in recent years. Pyroptosis is manifested by the continuous expansion of cells until the membrane ruptures, which leads to the release of cell contents and severe inflammation (Fang et al., 2020). Through comparing with other PCD, it is found that pyroptosis occurs more rapidly and is accompanied by plentiful pro-inflammatory factors (Xu et al., 2018), which constitutes the human natural immune barrier against exogenous infections and internal danger signals (Liang F. et al., 2020). Nowadays, growing small molecule substances and drugs that inhibit pyroptosis have been discovered, however, the amount of pyroptosis activator is rare (McKenzie et al., 2018; Sollberger et al., 2018; Yang J. et al., 2018; Hu et al., 2020; Humphries et al., 2020; Teng et al., 2020; Zhou et al., 2020a; Yu et al., 2021a) (Table 2). Besides, their applications are almost still in the cell research stage. Recent studies have shown that pyroptosis can participate in the process of various diseases, including tumors (Erkes et al., 2020; Zheng and Li, 2020), cardiovascular diseases (Zhaolin et al., 2019), atherosclerosis (AS) (Wu et al., 2018), diabetes (Tu et al., 2019) and so on. Therefore, an in-depth comprehension of the system about pyroptosis would be beneficial to the recognition and prevention of human diseases.

**TABLE 1:** The main characteristics of different types of cell death.

Types of Cell death	Morphological Changes	Canonical Initiators	Key Genes	Trigger Inflamma-Tion
Apoptosis	The cell shrinks, the chromatin and cell membrane concentrate, and the formation of apoptotic bodies	1) External initiators: TNF-α, FasL	Caspase- 2, 3,6,7, 8,9,10, Bax, Bak, Bcl-2, p53	No
		2) Internal initiators:		
		multiple stress, DNA damage		
		3) Endoplasmic reticulum stress		
Pyroptosis	The cell swells and deforms, the nucleus shrinks, DNA breaks, the cell membrane ruptures, and the cell contents release to cause a series of inflammatory reactions	Inflammation	Caspase-1,3.4,5,8,11, Gasdermin, NLRP1, NLRP3, IL-1β, IL-18	Yes
Necrosis	The cell volume increases, the organelle swells, the plasma membrane is damage, which eventually leads to cell ruptures, cell contents overflow to surrounding tissues, and the tissues are damaged	TNF-α, chemical or physical factors	RIP1, RIP3, MLKL	Yes
Ferroptosis	The cell membrane breaks and bubbles, the mitochondria becomes smaller, the mitochondrial membrane density increases and cristae decreases	Iron, glutamate	GPX4, p53, SLC7A11	Yes
			TFR1, IREB2, Nrf2, FSP1, ACSL4	
Autophagic cell death	The appearance of double membrane autophagosomes, then combine with lysosomes to form autolysosomes	Hunger, stress, energy metabolism	ATG genes, LC3, p62	No
Paraptosis	The cell membrane is intact, the cytoplasm is vacuolated, the mitochondria, the endoplasmic reticulum is swollen, and the nucleus do not shrink	IGF, insulin	ERK1/2, JNK1/2, p38	No

**TABLE 2:** Compounds and drugs that interfere with pyroptosis.

Compound	Effect	Mechanism	Experimental Cell Lines	References
Disulfiram	Inhibitor	Prevents GSDMD hole formation	mouse iBMDM/THP-1/HEK293T	Hu et al. (2020)
Ac-FLTD-CMK	Inhibitor	inhibits GSDMD cleavage by restraining caspases-1, -4, -5, and -11	RAW 264.7/HEK-293T/THP-1	Yang et al. (2018c)
LDC7559	Inhibitor	Inhibits	HEK-293T	Sollberger et al. (2018)
		GSDMD function and blocks IL-1β release		
Morroniside	Inhibitor	Inhibits cleaved caspase-1, NLRP3, and GSDMD	Primary mouse chondrocytes	Yu et al. (2021a)
Fumarate	Inhibitor	Inactivates GSDMD	BMDMs	Humphries et al. (2020)
HSP90 inhibitors	Inhibitor	Induce NLRP3 degradation through the proteasome	THP-1	Zhou et al. (2020a)
VX765	Inhibitor	Inhibits caspase-1 selectively	Microglial cells	McKenzie et al. (2018)
Polyphyllin VI	Activator	Induces NLRP3 inflammasome activation	A549/H1299	Teng et al. (2020)

The genetic central dogma states that the genetic information encoded in DNA should first be transcribed into messenger RNA (mRNA), and then translated into functional protein (Liu et al., 2018). Increasing studies have found that the maladjustment of the genetic central dogma will result in the occurrence of many diseases. Previous works have revealed that about 90% of the genes in the eukaryotic genome are transcribed genes. Of interest, only 1–2% of these transcribed genes encode proteins, most of them are transcribed into non-coding RNA (ncRNA) (Liu et al., 2021a). Although ncRNA lacks the potential to encode proteins, they can affect the expression of many molecular targets to drive specific cellular biological reactions and destinies. Therefore, ncRNA acts as key adjustment factor in physiological and pathological process. For example, ncRNA is particularly related to cancer, and have been identified as carcinogenic driving factor and tumor inhibitory factor in certain cancer (Anastasiadou et al., 2018). Furthermore, studies have indicated that ncRNA can affect cell fate decision (Schwarzer et al., 2017), as well as PCD (Su et al., 2016) *via* regulating gene transcriptional or post-transcriptional level. At present, it is shared for ncRNA to regulate apoptosis in various cells and tissues (Villanova et al., 2018; Hu et al., 2019; He et al., 2020), but the regulation of pyroptosis is remain uncovered. The exploration of ncRNA may be a new strategy of pyroptosis research, which is worthy of exhaustive analysis and clarification.

**FIGURE 1:**
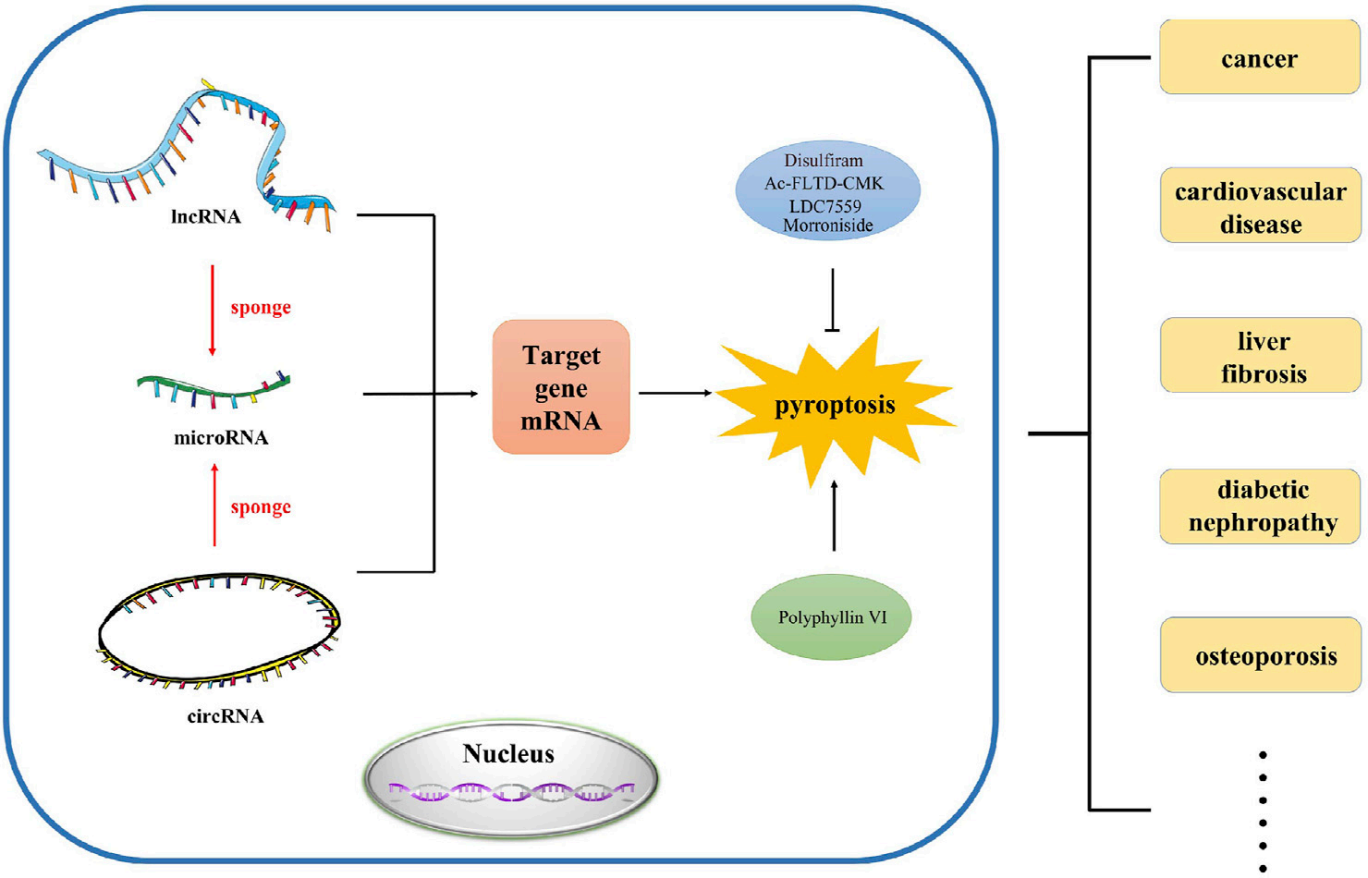
The abstract image of this review. In this review, we summarized the regulation and function of ncRNA such as lncRNA, microRNA and circRNA in pyroptosis, which provides novel research direction and strategy for the study of pyroptosis in different human diseases.

In this review, we mainly investigated the effects of various lncRNAs, miRNAs and circRNAs on pyroptosis in different diseases. In addition, we also revealed the role of ceRNA network in pyroptosis (Figure 1). This will facilitate the diagnosis and treatment of complex diseases.

## Core Mechanisms of Pyroptosis

**FIGURE 2:**
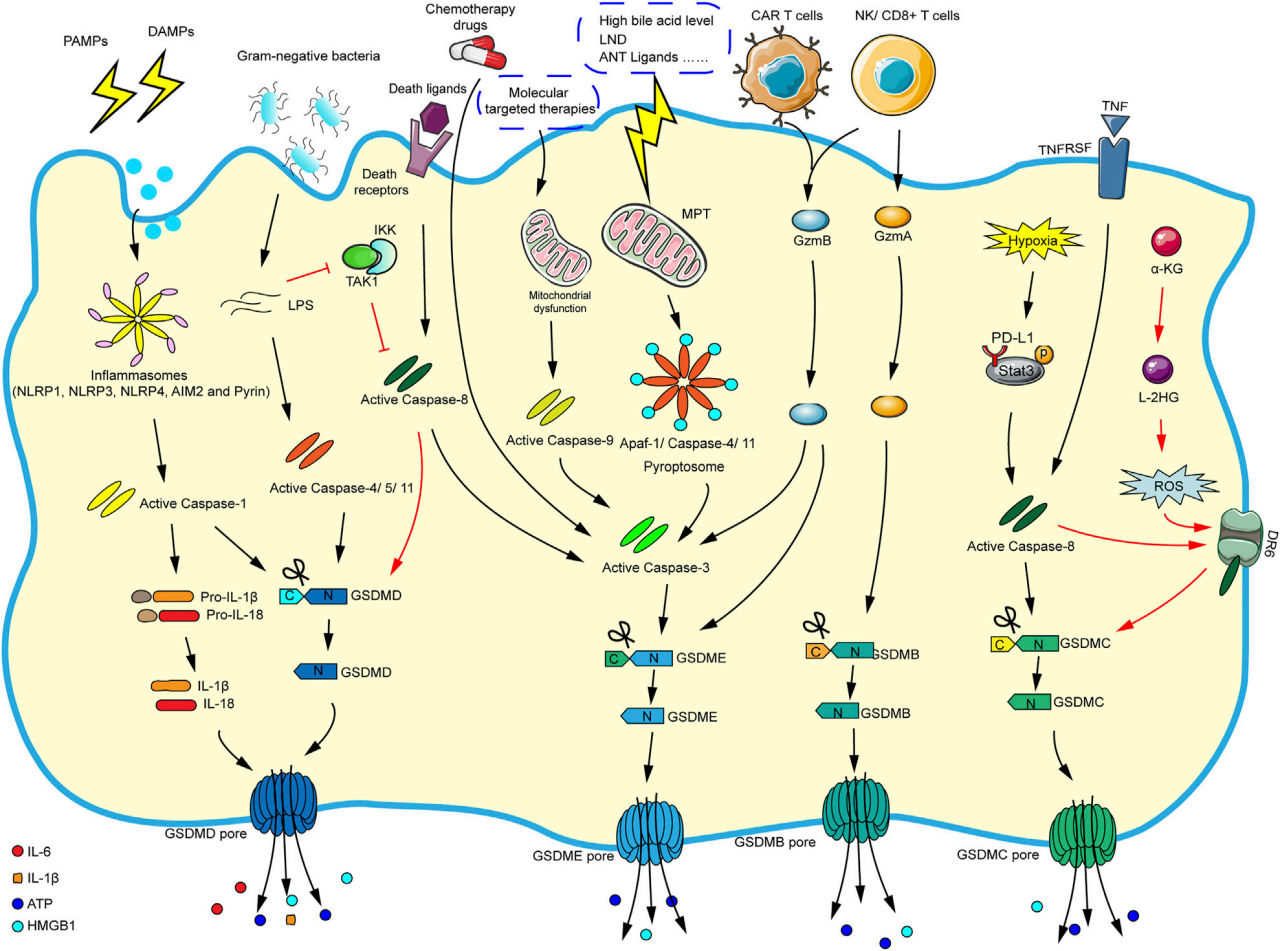
The main molecular mechanism of pyroptosis. In the caspase-1-dependent canonical inflammasome pathway, various inflammasomes are stimulated by cellular signals (such as PAMPs and DAMPs), which activate the inflammasomes and caspase-1. The activated caspase-1 cleaves GSDMD and pro-IL-1β/pro-IL-18, and finally mature IL-1β and IL-18 flow out of the GSDMD pore formed by the N-GSDMD oligomerization. In the caspase-4/5/11-dependent noncanonical inflammasome pathway, cytosolic LPS directly activates caspase-4/5/11, and the corresponding activated caspases will cleave GSDMD and eventually trigger pyroptosis. In the caspase-3-mediated pathway, chemotherapy drugs can directly activate caspase-3/GSDME-mediated pyroptosis. And molecular targeted therapies will elicit mitochondrial dysfunction and activate caspase-9, which eventually promote caspase-3/GSDME-mediated pyroptosis. In addition, the stimulation of high bile acid levels can result in MPT, then promoting Apaf-1/caspase-4/11 pyroptosome assembly, and ultimately causing caspase-3/GSDME-dependent pyroptosis. Caspase-3 can also be activated *via* caspase-8 when death ligands/receptors are stimulated. In the caspase-8-mediated pathway, in the response to LPS (such as Yersinia), the inhibition of TAK1/IKK complex activates caspase-8, and then triggers GSDMD-mediated pyroptosis. Moreover, under hypoxic conditions, PD-L1 binds to p-Stat3 in the nucleus, converting TNF-α-induced apoptosis into caspase-8/GSDMC-mediated pyroptosis. Besides, the metabolite α-KG can increase ROS production, promoting the assembly of DR6/pro-caspase-8/GSDMC receptosome, and activated caspase-8 cleaves GSDMC, leading to pyroptosis. In granzyme-mediated pathway, the CAR T cells rapidly activate caspase-3 by releasing GzmB, then GSDME-mediated pyroptosis is induced, and GzmB can directly act on GSDME. The GzmA derived by NK cells and lymphocytes could cleave GSDMB to result in pyroptosis.

Initially, research suggested that pyroptosis was related to the antibacterial response of immune cells (Sauer et al., 2010). So far, pyroptosis is usually induced by caspase-1-dependent canonical inflammasome and caspase-4/5/11-dependent noncanonical inflammasome pathways. It is worth noting that caspase-3 and caspase-8 (apoptosis-related caspase proteins) can also trigger pyroptosis. More attractively, some new mechanisms that induce pyroptosis have recently been discovered such as granzyme-mediated pyroptosis. We summarize the current mechanisms of pyroptosis in the Figure 2 according to the literatures.

### The Caspase-1-dependent Canonical Inflammasome Signaling Pathway

There are five main types of pattern recognition receptors (PRRs) involved in pathogen recognition: Toll-like receptors (TLRs), Nod-like receptors (NLRs), RIG-I-like receptors (RLRs), C-type lectin receptors (CLRs) and DNA sensors (Shekarian et al., 2019). Pathogen-associated molecular patterns (PAMPs) and damage associated molecular patterns (DAMPs) are two molecules closely related to innate immunity. When cells and tissues are damaged or under hypoxia condition, DAMPs are released into the blood circulation or intercellular spaces, while PAMPs are the molecular structures on pathogens. Both PAMPs and DAMPs are recognized by PRRs, which triggers immune response (Zindel and Kubes, 2020). The innate immune system is activated when PAMPs or DAMPs are burst, then the inflammasomes gradually formed in certain cells such as monocytes, macrophages and dendritic cells, because they can express more inflammation-related genes (Vande Walle and Lamkanfi, 2016). The canonical inflammasome is a polyprotein complex that contains a sensor protein, an adaptor protein named apoptosis-associated speck-like protein containing CARD (ASC), and an effector protein pro-caspase-1 (Man and Kanneganti, 2015; Rathinam and Fitzgerald, 2016). Currently, common sensor proteins mainly include NLRP1, NLRP3, NLRP6, NLRP12, NLRC4, AIM2, Pyrin, etc. In the canonical inflammasome-mediated pyroptosis pathway, once the sensor proteins receive signals, their oligomerization occurs rapidly, and the inflammasomes assembly are triggered, which leads to the polymerization of the adaptor protein ASC. Importantly, ASC is a double adaptor protein molecule which contains caspase recruitment domain (CARD) and pyrin domain (PYD). NLR containing PYD domain and pro-caspase-1 containing CARD domain are linked by ASC through the interaction of CARD-CARD and PYD-PYD. Followed by pro-caspase-1 in dimer form is cleaved into p10 and p20 subunits to form activated caspase-1, which induces the maturation of IL-1β and IL-18 from their precursors. In addition, activated caspase-1 cleaves and activates GSDMD to promote the occurrence of cell pyroptosis (Broz and Dixit, 2016; McKenzie et al., 2020). It seems that the formation of caspase-1-dependent canonical inflammasome is a key step in the driver of pyroptosis, and affecting the assembly and activation of inflammasome can be a classical way to regulate cell pyroptosis.

### The Caspase-4/5/11-dependent Noncanonical Inflammasome Signaling Pathway

In addition to the canonical inflammasome-mediated pyroptosis, noncanonical inflammasome can also trigger pyroptosis. The activation of inflammasomes usually follow one of these two pathways and depend on their activated caspases. The activation of canonical inflammasome depends on caspase-1, while noncanonical inflammasome activation is related to mouse-derived caspase-11 and human-derived caspase-4, 5. The Gram-negative bacteria (such as E. coli) can stimulate caspase-4, 5, 11 to induce the pyroptosis of macrophages (Muendlein et al., 2020). Bacterial LPS, a main immunogenic component of the outer leaflet of the cell wall of Gram-negative bacteria, is a necessary PAMP that gives rise to inflammation in Gram-negative bacterial infections (Matikainen et al., 2020). Recent findings suggest that in human epithelial cells guanylate-binding proteins 1 (GBP1) may act as a cytosolic LPS sensor and assembles a platform for caspase-4 recruitment and activation at LPS-containing membranes. It is regarded as the first step of non-canonical inflammasome signaling (Santos et al., 2020). When LPS leaks into the cytoplasm, it can directly bind to the CARD domain of caspase-11 (caspase-4, 5 in human) to induce its oligomerization and activation and then promote pyroptosis, interestingly, this process does not need to be mediated by activated caspase-1 (Vande Walle and Lamkanfi, 2016). Surprisingly, activated caspase-11 can also activate caspase-1 under certain circumstances (Aachoui et al., 2015). It can be seen that there is a crosstalk between caspase-1-dependent and caspase-11-dependent pyroptosis.

### The Caspase-3-Mediated Pathway

Notably, GSDME (also referred to as DFNA5) can also be cleaved and activated by caspase-3 to form soluble membrane pores. Studies have demonstrated that chemotherapeutic drugs can activate caspase-3-mediated cleavage of GSDME, thereby inducing pyroptosis (Wang et al., 2017). In addition, caspase-3/GSDME pathway is believed to participate in pyroptosis under caspase-9-mediated apoptosis stimulation (Lu et al., 2018). Apoptotic protease activating factor-1 (Apaf-1) plays a crucial role in apoptosis with the participation of mitochondria (Dorstyn et al., 2018). Of interest, Xu W. et al. (2021) discovered a new mechanism of caspase-3/GSDME-dependent pyroptosis, which is induced by Apaf-1/caspase-4/11 pyroptosome assembly activated through mitochondrial permeability transition (MPT). Attractively, this mechanism process is induced by bile acids rather than LPS stimulation. Further exploration determined that low-dose bile acids promoted mitochondrial outer membrane permeabilization (MOMP) and activated caspase-9-mediated apoptosis after a long time, while high-dose bile acids induced MPT and stimulated Apaf-1 more strongly, prompting Apaf-1 pyroptosome assembly selectively and caused pyroptosis. Can Apaf-1 trigger both pyroptosis and apoptosis in an intermediate state of mitochondrial stress? Hence, clarifying the dynamic changes of mitochondria may be the key point for the selection of different death pathways.

### The Caspase-8-Mediated Pathway

In the response to Yersinia infection, the activities of transforming growth factor β-activated kinase 1 (TAK1) and IκB kinase complex (IKK) are blocked, while the caspase-8 pathway is activated, which causing GSDMD to be cleaved and activated at the same location, finally, pyroptosis is triggered (Orning et al., 2018). Moreover, another finding indicate that in extrinsic and intrinsic apoptosis, caspase-1 and caspase-8 cleave GSDMD to trigger pyroptosis, importantly, extrinsic and intrinsic apoptosis both activate pannexin-1 to drive NLRP3 inflammasome assembly, further promoting pyroptosis (Chen K. W. et al., 2019). Among several kinds of caspase family, caspase-1 seems to be the strongest driving factor for GSDMD cleavage, and caspase-8 is the weakest driving factor. It is possible that caspase-8 acts as a backup measure when other caspase family members are damaged (Newton et al., 2019). Encouragingly, the researchers found that PD-L1 together with p-Stat3 transformed tumor cell apoptosis induced by TNFα into pyroptosis. More crucially, under hypoxic conditions, PD-L1 promoted GSDMC-mediated pyroptosis, which is specifically cleaved by caspase-8 rather than caspase-6 (Hou et al., 2020). Recent study suggests that in the acidic environment of tumor cells, the metabolic enzyme MDH1 reduces α-KG to L-2HG, increasing the level of reactive oxygen species (ROS) and inducing the oxidation and internalization of DR6 (the plasma membrane-localized death receptor), and then pro-caspase-8 and GSDMC are recruited to the internalized DR6 receptosome, which facilitates the activated-caspase-8 to cleave GSDMC, leading to pyroptosis ultimately (Zhang J.-y. et al., 2021).

### Granzyme-Mediated Pathway

Of interest, evidence demonstrated that chimeric antigen receptor (CAR) T cells rapidly activated caspase-3 in target cells by releasing granzyme B (GzmB), which cleaved GSDME and caused pyroptosis (Liu Y. et al., 2020). Shockingly, another study reported that GzmB could directly cleave GSDME to trigger pyroptosis, while caspase-3 was not indispensable for GzmB-mediated pyroptosis (Zhang Z. et al., 2020). GSDMB is highly expressed in certain tissues, especially the digestive tract epithelium. In 2020, Zhou et al. firstly discovered that natural killer cells (NKs) and cytotoxic T lymphoid (CTLs) cells killed GSDMB-positive cells *via* pyroptosis. The specific reason was that lymphocyte-derived granzyme A (GzmA) cleaved GSDMB at Lys229/Lys244 site (Zhou et al., 2020b).

### The Key Executioner of Pyroptosis: Gasdermin Family

Kayagaki et al. (2015), Shi et al. (2015) determined that GSDMD was the shared substrate protein of caspase-l, caspase-4, caspase-5 and caspase-11 almost at the same time. GSDMD is cleaved by caspase-1/4/5/11 to induce pyroptosis at the _272_FLTD_275_ site (_273_LLSD_276_ in mouse GSDMD). In addition, GSDMD is divided into an N-terminal p30 (GSDMD-N domain) and a C-terminal p20 (GSDMD-C domain). GSDMD-N can specifically bind to phosphorylated phosphatidylinositol or cardiolipin, which are specific phospholipids on eukaryotic and prokaryotic cell membranes respectively. GSDMD-N molecules bind to the cell membrane in this way and rapidly undergo oligomerization, and then 16 of these molecules form pores with an about 10–14 nm inner diameter. The inflammatory factors IL-1β, IL-18 and K^+^ produced *via* the inflammasome pathway are secreted out of the cell through these pores. At the same time, plenty of Ca^2+^ flows into the cell, resulting in increased osmotic pressure and electrochemical gradient, and a host of extracellular water enters the cell, causing the rupture and death of cell (Ding et al., 2016). Liu et al. (2017) have detected that NF-κB/GSDMD signaling is essential for the formation of NLRP3 inflammasome and the release of inflammatory cytokines during the melatonin inhibiting adipocytes pyroptosis. Furthermore, Kayagaki et al. (2019) indicated that in bone marrow–derived macrophages (BMDMs), interferon regulatory factor 2 (IRF2), a member of the transcription factors IRF family, could bind to regulatory elements in the GSDMD promoter to directly drive GSDMD transcription and trigger pyroptosis.

GSDMD belongs to the gasdermin family, which also includes GSDMA, GSDMB, GSDMC, GSDME and GSDMF (also referred to as DFNB59) (Tanaka et al., 2013). Except for GSDMF, all others have a pore-forming N-terminal domain and a C-terminal regulatory domain. These two domains are thought to be activated through cleavage and separation (Ding and Wang et al., 2016; Feng et al., 2018). In view of the death execution activity of the gasdermin family proteins, NCCD (The Nomenclature Committee on Cell Death) updated the definition of pyroptosis in 2018. The concept of pyroptosis is redefined as a regulated cell death (RCD) that is the formation of gasdermin family-dependent plasma membrane pores, and the inflammatory caspase activation is not indispensable. Therefore, a more comprehensive investigation of the mechanism and regulation of the gasdermin family will contribute to understand the pyroptosis mediated by gasdermin in the future, which will provide a theoretical basis for the treatment of pyroptosis-related diseases.

## Non-coding RNA and Pyroptosis

**FIGURE 3:**
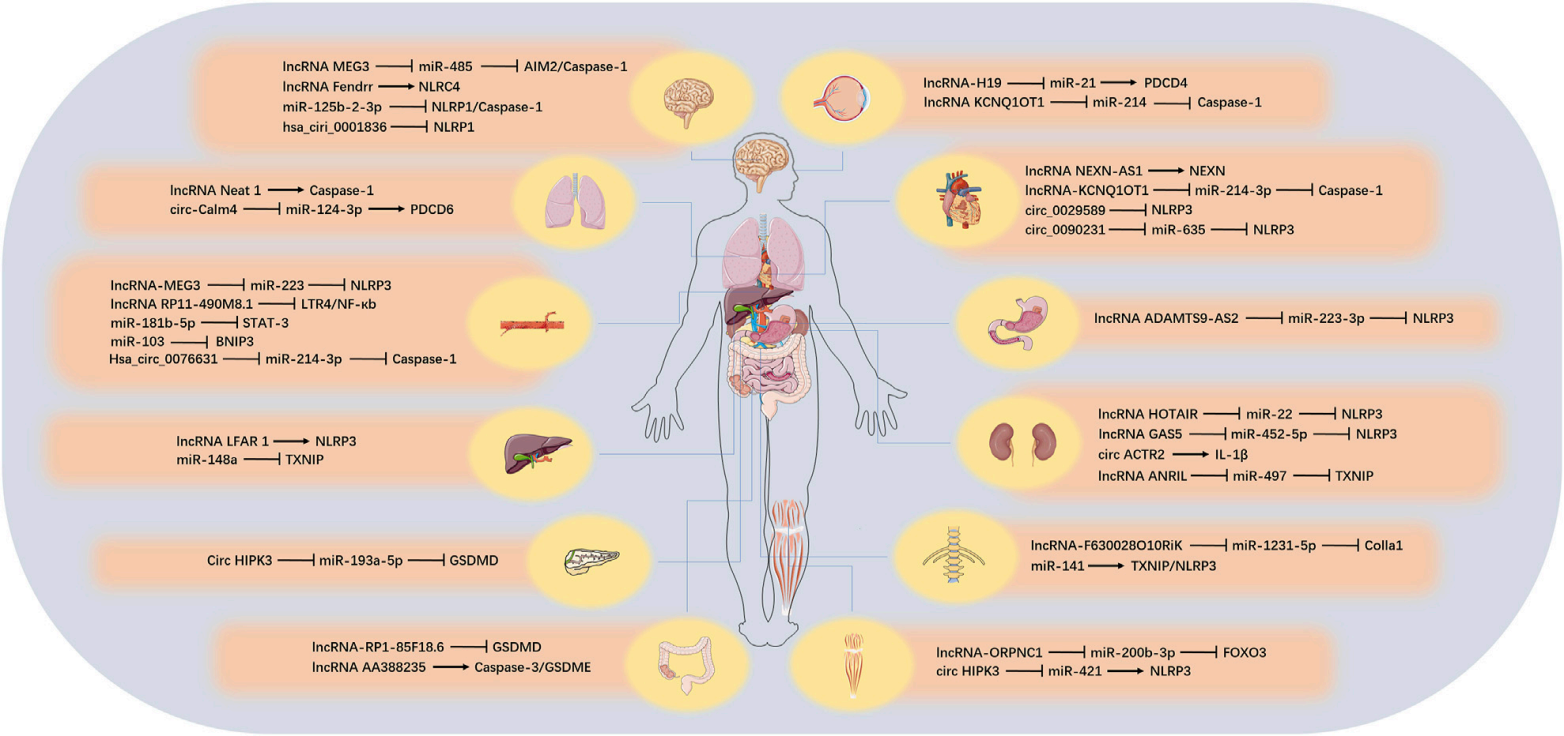
The role of ncRNAs on pyroptosis in different tissues. In different organs, various ncRNAs regulate the pyroptosis process by affecting the corresponding target genes.

There are numerous types of ncRNAs, which can be divided into two categories according to their length: those larger than 200 nt are called lncRNA, those smaller than 200 nt are called small ncRNA, and those below 50 nt can also be called tiny ncRNA (such as small interfering RNA [siRNA], miRNA, and [piwi-interactiing RNA] piRNA). In another way of classification, ncRNAs can also be divided into housekeeping ncRNA (such as ribosomal RNA [rRNA], transfer RNA [tRNA]) and regulatory ncRNA (such as miRNA, piRNA, lncRNA, tRNA-derived stress-induced RNA and tRNA-derived small RNA [tRF]) according to their expression and functional characteristics (Chen H. et al., 2019; Seal et al., 2020). The content of the housekeeping ncRNA is constitutive, which is essential for cell survival. The regulatory ncRNA is usually short-lived and regulates processes such as transcription and translation (Vivek and Kumar, 2021). Although ncRNA species are abundant, their roles in pyroptosis have been gradually revealed in recent years. Next, we reviewed the three regulatory ncRNAs involved in pyroptosis, including lncRNA, miRNA and circular RNA (circRNA) and the possible related mechanisms, and we also discussed the role of competing endogenous RNA (ceRNA) related to lncRNA and circRNA during pyroptosis process. These would provide a new research direction for the study of pyroptosis. We summarized the target, function, corresponding tissues and diseases of ncRNA involved in pyroptosis (Figure 3; Table 3).

**TABLE 3:** The function of ncRNAs involved in pyroptosis.

NcRNAs	Name	Target Genes	Effect of Pyroptosis	Related Diseases	References
LncRNAs	lncRNA-Neat1	caspase-1	+	pneumonia	Zhang et al. (2019)
	lnc-LFAR1	NLRP3	+	liver fibrosis	Zhang. et al. (2020a)
	lncRNA-NEXN-as 1	NEXN	−	atherosclerosis	Wu et al. (2020)
	lncRNA RP11-490M8.1	TLR4/NF-κb	−	atherosclerosis	Liu et al. (2021a)
	lncRNA-RP1-85F18.6	GSDMD	−	colorectal cancer	Ma et al. (2018)
	lncRNA AA388235	caspase-3/GSDME	+	colorectal cancer	Chen et al. (2021a)
	lncRNA-Fendrr	NLRC4	+	diabetic brain I/R damage	Wang et al. (2021a)
MiRNAs	miR-141	TXNIP/NLRP3	+	intervertebral disk degeneration	Xu et al. (2020a)
	miR-148a	TXNIP	−	alcoholic liver disease	Heo et al. (2019)
	miR-125b-2-3p	NLRP1	−	hypoxic-ischemic encephalopathy	Huang et al. (2020)
	miR-181b-5p	STAT-3	−	atherosclerosis	Xu et al. (2021a)
	miR-103	BNIP3	−	atherosclerosis	Wang et al. (2020b)
CircRNAs	hsa_circ_0001836	NLRP1	−	glioma	Liu et al. (2021b)
	circACTR2	IL-1β	+	diabetic kidney disease	Wen et al. (2020)
	circ_0029589	NLRP3	−	atherosclerosis	Guo et al. (2020a)
LncRNA-related ceRNA	lncRNA-H19/miR-21	PDCD4	+	retinal ischemia/reperfusion injury	Wen et al. (2020)
	lncRNA-F630028O10Rik/miR-1231-5P	Col1a1	+	spinal cord injury	Xu et al. (2020b)
	lncRNA-MEG3/miR-223	NLRP3	+	atherosclerosis	Zhang et al. (2018)
	lncRNA MEG3/miR-485	AIM2/caspase-1	+	cerebral ischemia-reperfusion injury	Liang et al. (2020b)
	lncRNA KCNQ1OT1/miR-214-3p	caspase-1	+	diabetes cardiomyopathy	Yang et al. (2018b)
	lncRNA KCNQ1OT1/miR-214	caspase-1	+	diabetic corneal endothelial keratopathy	Zhang et al. (2020b)
	lncRNA ADAMTS9-AS2/miR-223-3p	NLRP3	+	gastric cancer	Ren et al. (2020)
	lncRNA GAS5/miR-452-5p	NLRP3/caspase-1	−	diabetic nephropathy	Xie et al. (2019)
	lncRNA ANRIL/miR-497	TXNIP	+	diabetic nephropathy	Wang et al. (2021b)
	lncRNA-HOTAIR/miR-22	NLRP3	+	hyperuricaemia	Chi et al. (2021)
	lncRNA ORLNC1/miR-200b-3p	FOXO3	+	osteoporosis	Zhang et al. (2021b)
CircRNA-related ceRNA	circ_0090231/miR-635	NLRP3	+	atherosclerosis	Ge et al. (2021)
	circHIPK3/miR-421	NLRP3	−	ischemic muscle injury	Yan et al. (2020)
	circHIPK3/miR-193a-5p	GSDMD	+	acute pancreatitis	Wang et al. (2020b)
	hsa_circ_0076631/miR-214-3p	caspase-1	+	diabetic cardiomyopathy	Yang et al. (2019b)
	circ-Calm4/miR-124-3p	PDCD6	+	pulmonary hypertension	Jiang et al. (2021)

+: pyroptosis promotion; −: pyroptosis inhibition.

### LncRNA in Pyroptosis

LncRNA, transcript length exceeds 200 nt, is believed to have multiple functions, including cis or trans transcriptional regulation, nuclear domain organization and protein or RNA molecule regulation (Bridges et al., 2021). The functions are closely related to their subcellular location. In the nucleus, lncRNA regulates gene expression at the epigenetic and transcriptional levels, and in the cytoplasm, it influences gene expression at the post-transcriptional and translational spheres (Yang Z. et al., 2019). Dramatically, studies have also manifested that some transcripts such as lncRNA actually encode small proteins (Kopp and Mendell, 2018). It can be seen that the actual function of lncRNA has not yet been fully understood. Gradually, researchers paid attention to the regulation of lncRNA on cell fate. According to reports, lncRNA is involved in the regulation of cell death pathways such as apoptosis (Xiong et al., 2021), necrosis (Zhou et al., 2021), senescence (Montes et al., 2021)and autophagy (Zhou C. et al., 2020). Recently, the function of lncRNA on pyroptosis also has been continuously revealed.

lncRNA Neat1 is transcribed from a multitude of endocrine tumor formation sites. Findings (Zhang et al., 2019) demonstrated that in LPS-stimulated mouse immortal bone marrow-derived macrophages (iBMDM), lncRNA Neat1 boosted the activation of inflammasomes NLRP3, NLRC4 and AIM2, especially NLRP3, and enhanced caspase-1 activation to induce pyroptosis. Mechanistically, lncRNA Neat1 binds to pro-caspase-1 and promotes the assembly of inflammasomes. So that it can also stabilize mature caspase-1 and increase the activity of caspase-1 protease. According to research, lnc-LFAR1 is rich in liver without Kozak sequence, which is vital for the initiation of mRNA translation. Zhang et al. investigated that the silencing of lnc-LFAR1 alleviated the pro-inflammatory activation of M1 macrophages and restrained NLRP3 inflammasome-mediated pyroptosis stimulated by CCl_4_ and BDL ligation *in vivo*. Moreover, knockdown of lnc-LFAR1 also blocked LPS/ATP- and LPS/Niger-induced NLRP3 inflammasome-mediated pyroptosis *in vitro*. These data confirmed that lnc-LFAR1 played a crucial role in the containment of macrophage activation and pyroptosis, which provides an attractive therapeutic target for liver fibrosis. LncRNA NEXN-AS1 is a newly identified lncRNA with reduced expression in atherosclerotic plaques. It was found to interact with the nuclear protein BAZ1A to regulate its neighboring gene NEXN (Zhang K. et al., 2020). Wu et al. examined that atorvastatin could improve AS by inhibiting lncRNA NEXN-as1/NEXN-mediated pyroptosis. However, the research on lncRNA NEXN-AS1-mediated pyroptosis is still unclear, and it is worth exploring the underlying molecular mechanism (Wu et al., 2020). In another probe on AS (Liu X.-H. et al., 2021), researchers discovered that lncRNA RP11-490M8.1 blocked LPS-induced pyroptosis of Human Umbilical Vein Endothelial Cells (HUVECs) through the TLR4/NF-κb signal axis, implying that lncRNA RP11-490M8.1 may act as a therapeutic target for AS. Nevertheless, this work did not explore which gasdermin protein ultimately affected by lncRNA RP11-490M8.1, and did not identify it in-depth in animal models. Ma et al. indicated that the inhibition of lncRNA RP1-85F18.6 expression could accelerate the occurrence of colorectal cancer (CRC) cells pyroptosis by increasing the lysis of GSDMD (Ma et al., 2018). Not long ago, Chen et al. confirmed that siRNA designed for lncRNA AA388235, a kind of mouse-specific lncRNA, could induce the death of human CRC cells, yet had no effect on normal human cells. More attractively, the siRNA targeting mouse-specific lncRNA AA388235 could facilitate tumor cells pyroptosis through the caspase-3/GSDME signaling pathway, while in the absence of GSDME, tumor cells would undergo apoptosis (Chen Y.-R. et al., 2021). Therefore, species-specific lncRNA may be used as a sequence target to design siRNA for tumor treatment, which provides a new method for tumor drug design. Besides, researchers pointed out that in the diabetes-cerebral I/R model and microglia cells treated with high glucose followed by hypoxia/reoxygenation (H/R), lncRNA-Fendrr was highly expressed. LncRNA-Fendrr could prevent the ubiquitination and degradation of NLRC4 protein through the E3 ubiquitin ligase HERC2, thereby pushing the pyroptosis of microglia. This infers that lncRNA-Fendrr may be a potential target for the treatment of diabetic brain I/R damage (Wang L.-Q. et al., 2021). Above all, these studies not only further determine the vital role of lncRNA in pyroptosis, but also provide a fresh direction for the identification of disease markers.

### MiRNA in Pyroptosis

MiRNA is a type of small ncRNA with a length of approximately 21 nt (Xie et al., 2021). Foremost, the primary transcription product (pri-miRNA) of the miRNA gene is cleaved by RNase III Drosha in the nucleus to become the precursor miRNA (pre-miRNA). After the initial cleavage, pre-miRNA is transferred from the nucleus to the cytoplasm under the action of the transporter exportin-5, and then another Rnase III Dicer further cleaves it to produce mature miRNA. These mature miRNAs together with other proteins form a RISC (RNA-induced silencing complex) complex, which leads to the degradation of target mRNA or translation inhibition (Campbell et al., 2022). Recently, growing works have suggested that miRNA is largely involved in the pyroptosis, and it is of great significance to detect the role of miRNA in pyroptosis.

Xu et al. conducted microarray analysis on normal and degenerative nucleus pulposus (NP) and identified miRNA-141 as a key miRNA in intervertebral disk (IVD) degeneration. Further mechanism showed that miRNA-141 accelerated the ROS production and the activation of TXNIP/NLRP3 signaling pathway to induce nucleus pulposus cells (NPCs) pyroptosis along with extracellular matrix (ECM) catabolism (Xu Q. et al., 2020). Therefore, the miRNA-141-regulated pyroptosis may be a new therapeutic strategy for IVD degeneration. Evidences have implied that miRNA-148a have wealthy functions and is involved in prominent biological processes such as lipid metabolism (Cheng et al., 2017), inflammation (Humphries and Shmuel-Galia et al., 2020) and cell adhesion (Li et al., 2019). In the research of alcoholic liver disease (ALD), Heo et al. denoted that alcohol reduced the expression of miR-148a in hepatocytes by decreasing the expression of forkhead box protein O1 (FOXO1), and then promoted TXNIP expression and the activation of NLRP3 inflammasomes, thereby stimulating hepatocytes pyroptosis (Heo et al., 2019). The work provides a brand-new strategy for reducing the incidence of ALD. Huang et al. applied TargetScan, an RNA target prediction software, to conduct an online search. Results showed that miR-125b-2-3p contained a nucleotide sequence complementary to the highly conserved seed sequence in NLRP1 mRNA 3-UTR. Further exploration suggested that STF083010 (IRE1α inhibitor) could restrain neuronal pyroptosis regulated by miR-125b-2-3p/NLRP1 pathway in the neonatal hypoxic ischemic encephalopathy (HIE) rat model (Huang et al., 2020). Besides, miR-181b-5p has shear sensitivity in aortic valve endothelial cells and side specificity in porcine aortic valve respectively (Heath et al., 2018). Researchers in the AS study demonstrated that when HUVECs were induced by low shear stress, the expression of miR-181b-5p decreased, which increased STAT-3 expression and activated the NLRP3 inflammasome, eventually, the caspase-1-dependent pyroptosis was triggered (Xu X. et al., 2021). Thus, in-depth exploration of miR-181b-5p-mediated pyroptosis may be a promising way to treat AS. MiR-103 is widely expressed in various tissues, whose gene coding region is located on human chromosome 5 (Xu et al., 2015). Another outcome in AS indicated that miR-103 was reduced in H_2_O_2_-treated human coronary artery endothelial cells (HCAECs), which resulted in an up-regulation of Bcl-2/adenovirus E1B 19 kDa interacting protein (BNIP3) and the suppression of end-stage autophagy. As autolysosome synthesis is blocked, NLRP3 and caspase-1 increased, and cell pyroptosis was accelerated (Wang Y. et al., 2020). This phenomenon denoted that miR-103 might become a key point in the crosstalk between autophagy and pyroptosis, which offers an unusual basis for the regulation of pyroptosis. In general, these studies reveal the miRNAs regulate pyroptosis and expand the research field of pyroptosis.

### CircRNA in Pyroptosis

CircRNA that possesses regulatory functions has a closed loop structure and exists in an army of eukaryotic transcriptomes. Most circRNAs are derived from exons, and a small part is directly formed by introns cyclization. CircRNA is conserved in diverse species and it has specific expression in multiple tissues and different developmental stages. Besides, circRNA is divided into four categories, including exon circRNA (ecRNA), circular intron RNA (ciRNA), exon-intron circRNA (EIciRNA) and tRNA intron circular RNA (tricRNA) (Chen L. et al., 2021). Because circRNA is not sensitive to nucleases, it is more stable than linear RNA, which makes circRNA has obvious advantages in the development and application of new clinical diagnostic markers. CircRNA plays a pivotal role in various biological processes, such as autophagy (Liang G. et al., 2020), ferroptosis (Liu Z. et al., 2020), tumorigenesis (Guarnerio et al., 2019), tumor metabolism (Yu et al., 2019) and drug resistance (Liu J. et al., 2020). Amusingly, researchers have also presented new insights on circRNA in pyroptosis. Although there are a few reports in this area, the role of circRNA in pyroptosis is worthy of further detection.

Liu et al. analyzed that hsa_circ_0001836 was up-regulated in glioma cells, and its knockdown inhibited cell viability and proliferation. Engagingly, the reduction of hsa_circ_0001836 promoted pyroptosis of glioma cells. In mechanism, hsa_circ_0001836 knockdown may increase the expression of NLRP1 through DNA demethylation, thereby triggering cell pyroptosis (Liu et al., 2021c). The findings indicated that hsa_circ_0001836 might be a potential therapeutic target for the treatment of glioma. Wen et al. used circRNA chip analysis and found a series of dysregulated circRNAs in HK-2 cells stressed by glucose. Among the candidate circRNAs, circACTR2 was up-regulated and might be involved in inflammation and pyroptosis. Knockdown of circACTR2 could significantly reduce the production of interleukin (IL)-1β, the type IV collagen and fibronectin, even vitally blocked the occurrence of pyroptosis (Wen et al., 2020). The results manifested that circACTR2 could effectively regulate renal tubular cell pyroptosis, inflammation and fibrosis induced by high glucose. Whereas, the mechanism of circACTR2 in pyroptosis has not yet been elucidated, which is worthy of further clarification. Moreover, Guo et al. reported that IFN regulatory factor-1 (IRF-1) inhibited the pyroptosis and inflammation of macrophages in acute coronary syndrome (ACS) and AS *via* promoting the N6-methyladenosine (m6A) modification of circ_0029589 (Guo M. et al., 2020). The novel pyroptosis regulation mechanism provides great potentiality for the prevention and treatment of ACS and AS. Researches on circRNA in pyroptosis are limited, and further exploration is needed.

## CeRNA Cross-Talk in Pyroptosis

CeRNA is not a novel kind of RNA molecule, but a newly discovered regulatory mechanism. Recent studies have emphasized that genes have multiple modes of transcriptional regulation (Vivek and Kumar, 2021). One of the crucial regulatory factors, miRNA, can reverse-regulate gene expression by suppressing the translation of target genes or degrading target genes. In the actual regulation process, there is not only the simple miRNA-mRNA silencing mechanism, but also more complex regulatory network. Some ncRNAs also have binding sites with miRNA, which act as miRNA sponge in cells, thereby releasing miRNA. The inhibitory effect increases the expression level of target genes of miRNA, therefore a huge ceRNA network (ceRNET) is constructed. This mechanism of action is called the ceRNA mechanism (Li et al., 2021). CeRNA provides individuals with a new perspective for transcriptome exploration, which contributes to explain quite a few biological phenomena more comprehensively. To date, there has been growing learning on ceRNA, including cell pyroptosis. Underneath, we introduce the role of lncRNA and circRNA-related ceRNA in pyroptosis.

### LncRNA-Related ceRNA

A host of studies demonstrate that lncRNA participates in pyroptosis *via* coordinating with miRNA and protein-encoding mRNA. LncRNA acts as a ceRNA by competitively occupying the shared binding sequence of miRNA, thereby isolating miRNA and changing the expression of its downstream target genes. Such ceRNA networks formed by lncRNA/miRNA/mRNA interactions have been widely discovered in a multitude of biological processes, including epithelial-to-mesenchymal transition (EMT) (Yang X.-Z. et al., 2018), inflammation (Cao et al., 2020) and angiogenesis (Guo Z. et al., 2020). Below we summarize the quintessential examples of ceRNA networks related to lncRNA, which are involved in the underlying molecular mechanisms of pyroptosis.

LncRNA-H19 is one of the earliest discovered lncRNA and miR-21 exists widely in various organisms and mammalian tissues (Gamaev et al., 2021; Rossi et al., 2021). There is growing evidences have suggested that their regulatory effects are closely related to a variety of biological functions (Zhou et al., 2017; Morgoulis et al., 2019; Yu et al., 2021b). Wan et al. first used transcriptome sequencing technology to find that the level of lncRNA-H19 was obviously up-regulated during retinal ischemia/reperfusion (I/R) injury. Through further exploration, it was shown that lncRNA-H19 promoted programmed cell death protein 4 (PDCD4) expression by sponging miR-21 to form ceRNET, and then the balance of NLRP3/6 inflammasomes were regulated. Afterwards, caspase-1 was activated to cleave GSDMD and finally triggered microglia pyroptosis (Kai et al., 2020). This presents that the regulation of lncRNA-H19-related ceRNA-mediated pyroptosis may become a new idea to prevent I/R injury-related diseases. The research results of Xu et al. stated that during spinal cord injury (SCI), TLR4 was activated and promoted the expression of lncRNA-F630028O10Rik. This lncRNA acted as the ceRNA of the miR-1231-5p/Col1a1 axis and enhanced the microglia pyroptosis by activating the PI3K/AKT pathway after SCI (Xu S. et al., 2020). In the study of AS, Zhang et al. found that lncRNA MEG3 was enriched in endothelial cells and played a role of ceRNA on miR-223. Moreover, they proved that melatonin exerted an anti-pyroptosis effect by suppressing the lncRNA MEG3/miR-223/NLRP3 axis (Zhang et al., 2018). Surprisingly, another research denoted that lncRNA MEG3/miR-485/AIM2 axis promoted cell pyroptosis by activating caspase-1 signaling during cerebral I/R (Liang J. et al., 2020). Fan et al. treated cardiac fibroblasts with 30 mmol/L glucose, and the following results demonstrated that lncRNA KCNQ1OT1 acted as a ceRNA, which modulated the expression of caspase-1 by sponging miR-214-3p to trigger pyroptosis (Yang F. et al., 2018). Amusingly, in diabetic corneal endothelial keratopathy, the lncRNA KCNQ1OT1/miR-214 axis can also be found. Researchersreported that lncRNA KCNQ1OT1 promoted pyroptosis induced by high glucose through targeting miR-214/caspase-1 signaling (Zhang Y. et al., 2020). Ren et al. demonstrated that in cisplatin-treated gastric cancer (GC) cells, lncRNA ADAMTS9-AS2 activated NLRP3 inflammasome through sponging miR-223-3p to promote pyroptosis, which inhibited GC progress and made cisplatin-resistant GC (CR-GC) cells sensitive to cisplatin (Ren et al., 2020). The data offers new insights into the molecular mechanism of cisplatin resistance in GC cells, and is profitable to develop new clinical therapeutic drugs. In diabetic nephropathy (DN) study, Xie et al. pointed out that lncRNA GAS5 reduced the oxidative stress and renal tubular cells pyroptosis stimulated by high glucose. Mechanistically, lncRNA GAS5 prevented NLRP3-mediated pyroptosis through sponging miR-452-5p (Xie et al., 2019). This provides a new perspective for the treatment of. In another DN exploration, Wang et al. found that lncRNA ANRIL prevented TXNIP expression by acting as a sponge of miR-497, thereby giving rise to pyroptosis and kidney damage (Wang and Zhao, 2021). In high concentration of uric acid (HUA)-induced renal injury, Chi et al. proved that lncRNA-HOTAIR promoted endothelial cell pyroptosis by regulating the miR-22/NLRP3 signaling pathway (Chi et al., 2021). In addition, Zhang et al. also reported that lncRNA ORLNC1 induced the pyroptosis of bone marrow mesenchymal stem cells (BMSCs) induced by CML [Nε-(carboxymethyl) lysine, the most common advanced glycation end-products (AGEs)] through targeting the miR-200b-3p/FOXO3 axis (Zhang L. et al., 2021). The above researches confirmed that lncRNA could be used as ceRNA to participate in the process of pyroptosis, which broaden horizons for the pyroptosis exploration.

### CircRNA-Related ceRNA

Currently, increasing reports have denoted that circRNA-related ceRNA is involved in physiological process. CircRNA can compete with miRNA to affect the stability of target RNA or their translation, thereby regulating gene expression at the transcriptional level (Wang L. et al., 2021). Evidences suggested that circRNA can be used as ceRNA to participate in biological processes, such as tumor cell proliferation (Cheng et al., 2019), apoptosis (Sang et al., 2019), invasion (Luo et al., 2020) and migration (Wang et al., 2019). It is worth noting that the function of circRNA as ceRNA in pyroptosis has also been continuously presented.

Ge et al. treated human aortic endothelial cells (HAECs) with oxidized low-density lipoprotein (ox-LDL) to construct the AS model. They found that circ_0090231 promoted cell pyroptosis *in vitro*. What’s more, they identified circ_0090231 as a sponge of miR-635, and its knockdown reversed the effect of circ_0090231. Finally, it showed that the circ_0090231/miR-635/NLRP3 axis affected the development of AS by regulating cell pyroptosis (Ge et al., 2021). Yan et al. indicated that circHIPK3 derived from stem cell-derived exosomes could interact with miR-421 to regulate the expression of FOXO3a, resulting in the down-regulation of NLRP3 and caspase-1, ultimately inhibiting the pyroptosis of skeletal muscle cells and enhancing the repair of ischemic hind limbs (Yan et al., 2020). Dramatically, another study also hinted that the role of circHIPK3 in acinar cells pyroptosis. They demonstrated that circHIPK3 promoted pyroptosis by regulating the miR-193a-5p/GSDMD axis (Wang J. et al., 2020). Obviously, the roles of circHIPK3 in pyroptosis are opposite in the two reports. In the exploration of diabetic cardiomyopathy (DCM), Yang et al. emphasized that hsa_circ_0076631, named caspase-1-associated circRNA (CACR), was significantly increased in the serum of diabetic patients, and the silencing of CACR could reduce caspase-1 expression by sponging miR-214-3p, thereby restraining inflammation and pyroptosis of cardiomyocytes (Yang F. et al., 2019). Jiang et al. proved that in hypoxia-induced pulmonary arterial smooth muscle cells (PASMCs), circ-Calm4 increased the expression of programmed cell death protein 6 (PDCD6) through sponging miR-124-3p, and ultimately triggered pyroptosis (Jiang et al., 2021). Although the current research on circRNA-related ceRNA in pyroptosis is insufficient, it is believed that the achievements in this field would be even greater in over time.

## Potential Clinical Significance of the Regulation Between ncRNA and Pyroptosis

Overall, this review reveal how ncRNAs accurately regulate the cell pyroptosis processes in different tissues or cells. The above findings show that although ncRNAs lack the potential of proteins, they can regulate pyroptosis by a variety of mechanisms. Increasing researches have stated that ncRNAs can be used to predict the diagnosis and prognosis of cancer or other diseases (Rincon-Riveros et al., 2021). Perhaps ncRNAs can also become pivotal indications of pyroptosis-related diseases. Then the expression of the pyroptosis-related genes can be adjusted through acting on the consensus sequence of ncRNAs specifically. Therefore, various ncRNAs, as potential targets, can provide new possibilities for clinical treatment by affecting pyroptosis. However, the specific mechanisms of targeting ncRNA need to be further confirmed.

## Conclusion and Future Perspectives

Since the emergence of pyroptosis, it has aroused strong interest of researchers, and reached a lot of achievements. Evidences have confirmed that pyroptosis is closely related to many diseases, such as tumors (Erkes et al., 2020; Zheng and Li, 2020), cardiovascular diseases (Zhaolin et al., 2019), AS (Wu et al., 2018) and diabetes (Tu et al., 2019). In some cases, pyroptosis releases the cytoplasmic contents of dying host cells, thereby providing a powerful signal to initiate the inflammatory cascade. Local inflammation leads to the recruitment and activation of immune cells, which ultimately helps the host clear pathogens. Even so, in the context of chronic autoinflammatory diseases and sepsis, uncontrolled pyroptosis is extremely destructive. This raises a series of questions: Which pathological conditions will cause cell pyroptosis? When pyroptosis should be suppressed or promoted? Where are the inflammatory factors released from pyroptosis cells going? These are all paramount issues that need to be resolved urgently. Nevertheless, the analysis of the pyroptosis mechanism and its exact role in the body still remain uncovered.

Notably, the exploration of ncRNA has achieved many gratifying outcomes, involving the process of a variety of diseases, such as liver fibrosis, kidney damages, tumors and neurodegenerative diseases. What is remarkable is that increasing evidences show that ncRNA plays a pivotal role in pyroptosis, which opens up a novel way for the pyroptosis mechanism. However, due to the wide varieties of ncRNA, it is arduous to explore the specific mechanism of ncRNA in pyroptosis. Obviously, there are various differentially expressed ncRNAs-induced pyroptosis in the same disease. At present, it is still difficult to determine which ncRNA is the most effective intervention method of pyroptosis. Furthermore, how to trigger specifically the abnormal cells (such as tumor cells) pyroptosis by regulating ncRNA is still facing a huge challenge. Another serious problem is that different cell death modes such as pyroptosis, apoptosis and autophagy might be under the control of the same ncRNA. How does ncRNA keep a balance between pyroptosis and other death pathways requiring more in-depth exploration. The influence of ncRNA on pyroptosis is complex, especially in different cells and diseases. How to exactly intervene different ncRNAs-mediated pyroptosis still requires more experiments to investigate.
